# Decreased Triacylglycerol Content and Elevated Contents of Cell Membrane Lipids in Colorectal Cancer Tissue: A Lipidomic Study

**DOI:** 10.3390/jcm9041095

**Published:** 2020-04-12

**Authors:** Adriana Mika, Alicja Pakiet, Aleksandra Czumaj, Zbigniew Kaczynski, Ivan Liakh, Jarek Kobiela, Adrian Perdyan, Krystian Adrych, Wojciech Makarewicz, Tomasz Sledzinski

**Affiliations:** 1 Department of Environmental Analytics, Faculty of Chemistry, University of Gdansk, Wita Stwosza 63, 80-308 Gdansk, Poland; alicja.pakiet@phdstud.ug.edu.pl; 2 Department of Pharmaceutical Biochemistry, Faculty of Pharmacy, Medical University of Gdansk, Debinki 1, 80-211 Gdansk, Poland; aleksandra.czumaj@gumed.edu.pl (A.C.); liakh_ivan@mail.ru (I.L.); tsledz@gumed.edu.pl (T.S.); 3 Faculty of Chemistry, University of Gdansk, Wita Stwosza 63, 80-308 Gdansk, Poland; zbigniew.kaczynski@ug.edu.pl; 4 Department of Endocrine and Transplant Surgery, Faculty of Medicine, Medical University of Gdansk, Smoluchowskiego 17, 80-214 Gdansk, Poland; kobiela@gumed.edu.pl; 5 Department of Pathomorphology, Faculty of Medicine, Medical University of Gdansk, Smoluchowskiego 17, 80-214 Gdansk, Poland; 532at@gumed.edu.pl; 6 Department of Gastroenterology and Hepatology, Faculty of Medicine, Medical University of Gdansk, Smoluchowskiego 17, 80-214 Gdansk, Poland; krystian.adrych@gumed.edu.pl; 7 Department and Clinic of Surgical Oncology, Faculty of Medicine, Medical University of Gdansk, Smoluchowskiego 17, 80-214 Gdansk, Poland; wojmakar@wp.pl

**Keywords:** colorectal cancer, lipids, polyunsaturated fatty acids, cancer cell proliferation, nuclear magnetic resonance, cell membrane, lipid oxidation

## Abstract

Recent evidence suggests that lipid composition in cancer tissues may undergo multiple alterations. However, no comprehensive analysis of various lipid groups in colorectal cancer (CRC) tissue has been conducted thus far. To address the problem in question, we determined the contents of triacylglycerols (TG), an energetic substrate, various lipids necessary for cell membrane formation, among them phospholipids (phosphatidylcholine, phosphatidylethanolamine), sphingolipids (sphingomyelin) and cholesterol (free, esterified and total), and fatty acids included in complex lipids. ^1^H-nuclear magnetic resonance (^1^H-NMR) and gas chromatography-mass spectrometry (GC-MS) were used to analyze the lipid composition of colon cancer tissue and normal large intestinal mucosa from 25 patients. Compared with normal tissue, cancer tissues had significantly lower TG content, along with elevated levels of phospholipids, sphingomyelin, and cholesterol. Moreover, the content of oleic acid, the main component of TG, was decreased in cancer tissues, whereas the levels of saturated fatty acids and polyunsaturated fatty acids (PUFAs), which are principal components of polar lipids, were elevated. These lipidome rearrangements were associated with the overexpression of genes associated with fatty acid oxidation, and the synthesis of phospholipids and cholesterol. These findings suggest that reprogramming of lipid metabolism might occur in CRC tissue, with a shift towards increased utilization of TG for energy production and enhanced synthesis of membrane lipids, necessary for the rapid proliferation of cancer cells.

## 1. Introduction

Colorectal cancer (CRC) is one of the most common malignancies worldwide, and its incidence is still increasing [1,2,3,4]. Prevention, treatment and prognosis in CRC cannot be improved any further without a better insight into the pathophysiology of this malignancy [1].

Although lipidomic studies can provide information about the biochemical role of lipids and their exact physiological functions, the problem is quite complex [5]. Based on the comparison of lipid metabolism in various physiological and pathological conditions, identification of critical structures, their functions and interactions with other lipids, proteins, and different metabolites, lipidomics may explain the role of these compounds in metabolic control [1]. Given the important role of lipids at various levels of cell physiology, changes in their metabolism may interfere with a variety of processes, e.g., the control of the plasticity and organization of plasma membranes, or the provision of substrates for ATP synthesis and cell signaling [3]. Some authors have claimed a positive effect of supplementation with various lipids [2,6] or their main structural elements, fatty acids (FAs), in cancer patients [7]. However, the effects of FAs were also shown to depend on the structural group they belong, as well as on their source (de novo synthesis, diet) [3]. 

The results of some observational studies suggest that dietary provision of n-3 polyunsaturated FAs (PUFAs) from marine food sources might mitigate CRC risk and reduce mortality associated with this malignancy [8]. However, other authors found no link between n-3 and n-6 PUFAs and CRC development or treatment outcomes [9]. Furthermore, the available evidence in this matter is insufficient, as most preclinical studies analyzed the direct effects of selected n-3 PUFAs on cancer cells in vitro, rather than in vivo [10]. Some evidence suggests that potential therapeutic effects of n-3 PUFAs, if any, may depend on their dose, concomitant chemotherapy and its regimen [11]. Moreover, still little is known about lipid and FA composition in CRC cells. Lipids are considered as a potential risk or severity marker in many malignancies, including breast cancer [12,13], prostate cancer [14], ovarian cancer [15], lung cancer [16], pancreatic cancer [17], and bladder cancer [8,12]. Furthermore, lipids were postulated to be a potential therapeutic target and a basis for novel treatment strategies [1,8]. Some researchers reported changes in the FA composition of various lipid fractions and total lipids in CRC tissues [8,18,19,20], as well as in plasma, serum, erythrocytes [8,21], and adipose tissue from patients with this malignancy [8]. However, to the best of our knowledge, the exact composition of various lipid groups and FA profile have not been analyzed comprehensively in CRC thus far.

In view of published evidence, determination of the lipid composition of cancer tissue and understanding of mechanisms underlying the differences in the lipid profiles of normal and cancer tissues seem to be vitally important. Despite revealing the biological effects of multiple lipid groups, determination of lipid metabolism and function in cancer tissue still constitutes a challenge [1]. In this study, we used ^1^H-nuclear magnetic resonance (^1^H-NMR) to comprehensively analyze the composition of various lipid groups in CRC and normal tissues. Additionally, we determined the fatty acid composition of these tissues using gas chromatography-mass spectrometry (GC-MS).

## 2. Materials and Methods

### 2.1. Patients

**Figure 1:**
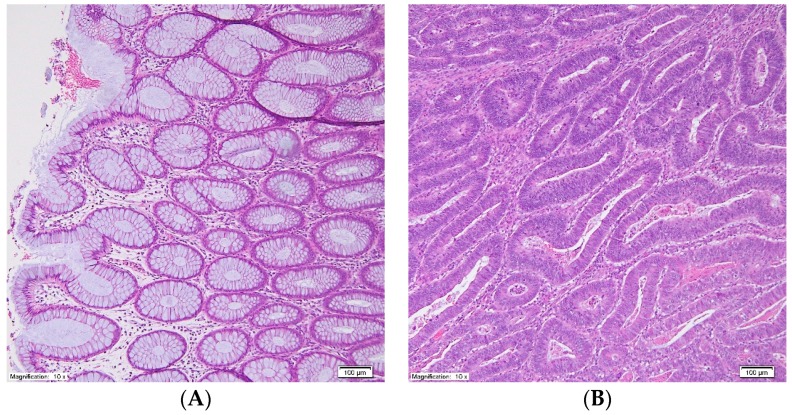
Representative photographs of normal large intestinal mucosa (**A**) and colorectal cancer tissue (**B**) after hematoxylin-eosin stain, original magnification: 10×.

The study included serum and tissue samples from 25 patients (16 men and nine women). The patients were operated on for stage I to stage IV CRC. None of the patients received preoperative neoadjuvant treatment. Biochemical and clinical characteristics of the study patients are presented in Table 1. Tissue samples were collected from the tumor and normal large intestinal mucosa, at least 5 cm from the tumor interface. Most cancers found in the colon or rectum are adenocarcinomas arising from pathological lesions in the epithelial cells of colorectal mucosa [22]. Thus, normal colon mucosa is a commonly used control for CRC [23,24,25]. The tissue was collected during surgery. Each sample was divided into two parts. The representative photographs of both tissues after H&E staining are presented on Figure 1. The part designated for the lipidomic study was frozen in liquid nitrogen immediately after collection and stored in aliquots at −80 °C until the analysis. The other part was used for histopathological examination. During histological analysis of cancer tissue the following features were determined: % of epithelial cells in tumor and % of stroma in tumor (within stroma: % of connective tissue cells and % of inflammatory cells). Microscopic evaluation was done in routinely H&E stained samples. The field with the most cancerous tissue density was examined under the tenfold zoom. Areas of necrosis and lumen of tubular malignant structures were excluded from estimated microscopic picture, thus percentages of epithelial cells in tumor and stroma in tumor do not add up to 100%. On the other hand, percentages of connective tissue cells and inflammatory cells add up to 100% since they are components of the tumor’s stroma. 

Moreover, 5-mL fasting blood samples were collected from all CRC patients. The samples were collected into tubes without anticoagulant, left at room temperature for 30 min to allow clotting and centrifuged at 3000× *g* for 15 min at 4 °C. After the centrifugation, the serum samples were stored in aliquots at −80 °C until the analysis. The protocol of the study was compliant with the Declaration of Helsinki of the World Medical Association and was granted approval from the Local Bioethics Committee at the Medical University of Gdansk (decision no. NKBN/487/2015). Written informed consent was obtained from all the patients prior to the study. Serum glucose, triglyceride, HDL, LDL, total cholesterol, hs-CRP, albumin, and total protein levels were determined with XL-100 analyzer (Erba Diagnostics Mannheim GmbH, Mannheim, Germany).

### 2.2. Lipid Sample Preparation for ^1^H-NMR and GC-MS Analyses

Total lipids were extracted from tissue samples (about 150 mg) with a chloroform-methanol mixture (2:1, v/v), according to the Folch et al. method [26], and dried under a nitrogen stream.

### 2.3. ^1^H-NMR Spectroscopic Analysis

Prior to NMR analysis, the 2 mg of extracted lipids were dissolved in 600 μL of deuterated chloroform-deuterated methanol mixture (2:1, v/v), and 3 mM of tetramethylsilane (TMS) was added as an internal standard. The samples were centrifuged and transferred to 5-mm NMR tubes. The NMR spectra for lipid extracts were recorded at 298 K, using a Brucker Avance III 500 MHz (Billerica, MA, USA) with TMS signal as a reference (0.00 ppm). The ^1^H-NMR spectra were recorded with zg30 pulse sequence. A total of 64 subsequent scans were collected for each sample, with an acquisition time of 4 s, spectral width of 15 ppm, and FID size of 65K. 

#### NMR Data Processing

The NMR spectra were processed with a line broadening of 0.3 Hz and manually phased and baseline-corrected using MestReNova software (Mestrelab Research v. 11.0, MestReLab Research S.L., Santiago de Compostela, Spain). The spectra were integrated manually, and all integrals were scaled to the TMS reference signal. The signals’ intensities were then normalized on 1 mg of lipids.

### 2.4. GC-MS Analysis

Lipid extracts were hydrolyzed with 0.5 M KOH in methanol at 90 °C. After 3 h, the samples were acidified with 0.5 mL 6 M HCl and added 1 mL of water. Fatty acids were extracted three times with 1 mL of n-hexane and dried under a nitrogen stream. Fatty acid methyl esters (FAMEs) were prepared by adding 0.5 mL of 10% boron trifluoride in methanol solution and incubating at 55 °C for 1.5 h. Then, the FAMEs were extracted thrice with n-hexane as described above and dried under a nitrogen stream. The analysis was carried out using GC-EI-MS QP-2010SE (Shimadzu, Kyoto, Japan). FAMEs were separated on Zebron ZB-5MSi capillary column (30 m length × 0.25 mm i.d. × 0.25 µm film thickness) (Phenomenex, Torrance, CA, USA), with oven temperature set at 60–300 °C, 4 °C/min ramp rate, and 60-min overall runtime. Helium was used as the carrier gas, with column head pressure of 100 kPa. The acquisition of mass spectra was carried out in full-scan mode, with the mass scan range set at m/z 45–700 and electron impact source operating at 70 eV. 19-methylarachidic was added as an internal standard. Fatty acids were identified against known reference standards (37 FAME Mix, Sigma-Aldrich, St. Louis, MO, USA) and reference library NIST 2011.

### 2.5. RNA Isolation and Real-Time Analysis of mRNA Levels

Total RNA isolation from CRC and normal tissue samples (about 50 mg) was carried out with RNeasy Plus Universal Kit (Qiagen, Hilden, Germany), in accordance with the manufacturer’s protocol. A NanoDrop One spectrophotometer (ThermoFisher Scientific, Waltham, MA, USA) was used to analyze the RNA concentration and purity. All samples were DNase-treated (DNase I, RNase-free, ThermoFisher Scientific) to remove any contaminating DNA. For PCR amplification, the first-strand cDNA was synthesized from 1 μg of total RNA, using random hexamer primers and RevertAid First Strand cDNA Synthesis Kit (ThermoFisher Scientific, Waltham, MA, USA). Oligonucleotide primers were designed for the specific amplification of selected enzymes which catalyze the rate-limiting reactions of FA transport into mitochondria, that regulates the rate of β-oxidation (carnitine palmitoyltransferase I) and in the synthesis of cholesterol, (HMG-CoA reductase), phosphatidylcholine (choline phosphotransferase 1), sphingomyelin (sphingomyelin synthase 1 and 2), and phosphatidylethanolamine (phosphatidylserine decarboxylase). PCR amplification was carried out with a CFX Connect Real-Time PCR Detection System (Bio-Rad, Hercules, CA, USA). Expressions of target genes were calculated based on the delta-delta Ct method. Housekeeping β-actin gene was used for normalization. The sequences of primers are presented in Table S1.

### 2.6. Statistical Analysis

Statistical significance of differences in the study parameters was verified with paired Student t-test (cancer tissue vs. normal colorectal mucosa) for data with normal distribution or Wilcoxon signed-rank test for data with not normal distribution. The differences were considered significant at *p* < 0.05. Relationships between pairs of variables were determined on the basis of linear regression analysis. The data from all 25 patients were included in all the analysis. The results are presented as means ± standard error of the mean (SEM). All statistical calculations were carried out with SigmaPlot software (Systat, S oftware Inc, San Jose, CA, USA). The multivariate analyzes were performed using MetaboAnalyst 4.0 (Montreal, Canada) [27].

## 3. Results

The epithelial component was larger than tumors’ stroma, and within stroma connective tissue cells were more numerous than the inflammatory cells (Table 1). The cancer epithelial and stroma cells were absent in all samples of normal tissue.

**Figure 2:**
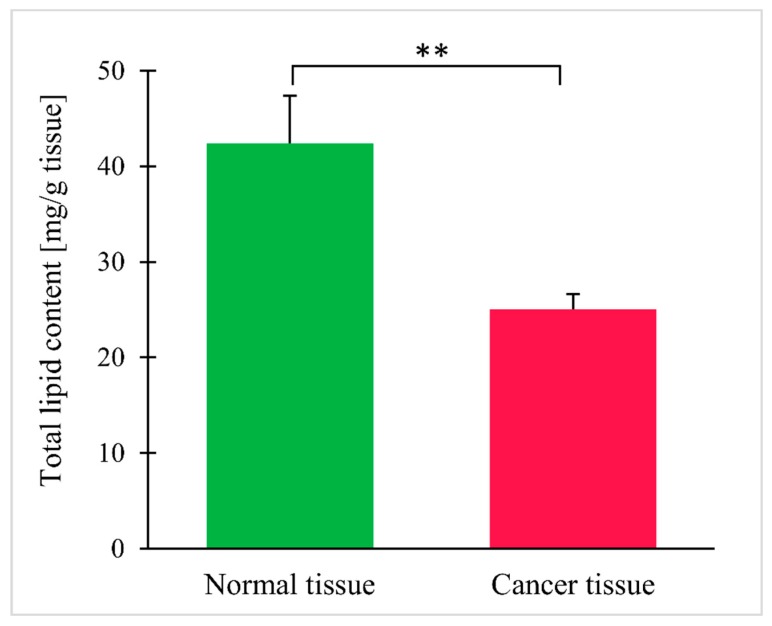
Total lipid content in normal and colorectal cancer tissue. Values are mean ± SEM; ** *p* < 0.01, *n* = 25.

Total lipid content in cancer tissue turned out to be lower than in normal tissue (Figure 2). The results for each individual patient are shown in the supplementary file (Figure S1). 

**Figure 3:**
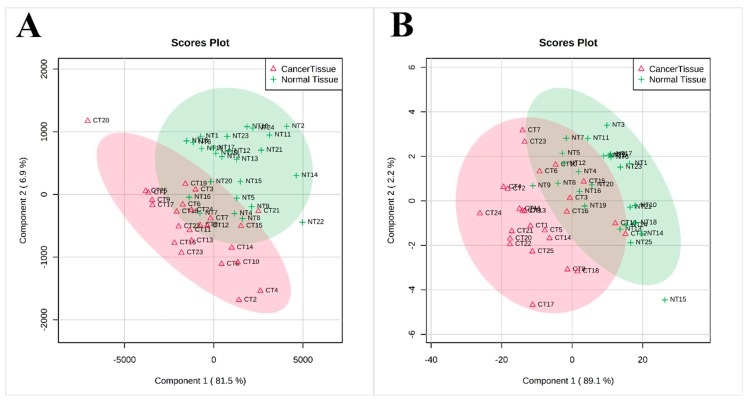
PLS-DA scores scatter plots obtained for normal and cancer tissue ^1^H-NMR spectra (**A**) and GC-MS analysis (**B**); *n* = 25.

Than we performed a multivariate analysis of data obtained by ^1^H-nuclear magnetic resonance (^1^H-NMR) and GC-MS to see if there is a difference in lipid groups and FA composition between normal and cancer tissues. Figure 3 shows the results of partial least square discriminant analysis (PLS-DA), which revealed the separation between the normal and cancer tissue based on lipid groups assayed by NMR (Figure 3A) or FA content assayed by GC-MS (Figure 3B). 

**Figure 4:**
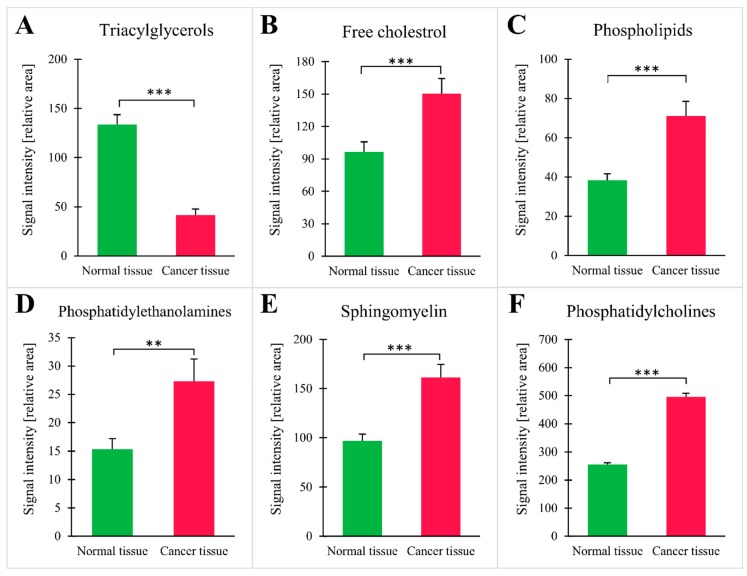
Intensities of NMR signal for selected groups of lipids: (**A**) triacylglycerols; (**B**) free cholesterol; (**C**) phospholipids; (**D**) phosphatidylethanolamines; (**E**) sphingomyelin; (**F**) phosphatidylcholines; values are mean ± SEM; ** *p* < 0.01 *** *p* < 0.001, *n* = 25.

When ^1^H-NMR was applied, a total of 26 lipid groups were identified, with statistically significant differences in signal intensities for CRC and normal tissue (Table 2). An example of a ^1^H-NMR spectrum for lipids isolated from normal tissue and CRC is presented in the supplementary file (Figure S2). The analysis at the lipid group level demonstrated that cancer tissue contained fewer triacylglycerols (TG), the main lipid energy depot in the cell (Figure 4A). Moreover, cancer tissue had higher levels of membrane lipids, such as free cholesterol, phospholipids, phosphatidylcholine (PC), phosphatidylethanolamine (PE), and sphingomyelin (SM) (Figure 4B–D). The results for each individual patient are shown in supplementary file (Figure S3). The patients taking statins (*n* = 7) tended to have lower signal of free cholesterol than the rest of patients (111 ± 24 vs. 172 ± 20), but this difference did not reach statistical significance (*p* = 0.08). The analysis in this context was underpowered. 

Compositions of FAs in cancer tissue and normal large intestinal tissue were determined using gas chromatography-mass spectrometry (GC-MS). Contents of various FA groups and major fatty acids from each group are presented in Table 3. Compared with normal tissue, CRC tissue was shown to contain significantly less MUFAs and oleic acid (18:1), being major components of TGs. Furthermore, CRC tissue had slightly higher levels of SFAs and considerably higher levels of n-3 and n-6 PUFAs than the normal tissue (Table 3). Compared with normal tissue, the levels of eicosapentaenoic acid (20:5n-3, EPA) and docosahexaenoic acid (22:6n-3, DHA) in cancer tissue were two-fold higher, whereas the levels of arachidonic acid (20:4n-6, ARA) was 2.5-fold higher. However, no significant difference in the precursor of ARA, linoleic acid (18:2n-6, LA), was found between CRC and normal tissue, and there was slight, but significant difference between normal and cancer tissue in α-linolenic acid (18:3n-3, ALA), a precursor of other n-3 PUFA. Finally, the level of one SFA, stearic acid (18:0) in CRC tissue was nearly twice as high as in normal tissue (Table 3).

We have also performed an analysis of correlations between tissue lipid groups, fatty acids and serum TG, total cholesterol and BMI. Moreover, we searched for differences in lipids according to stage of disease and sex. However, we did not find any significant correlations or differences, perhaps due to the relatively small cohort of this study. These results are presented as a Supplementary Tables S2 and S3.

**Figure 5:**
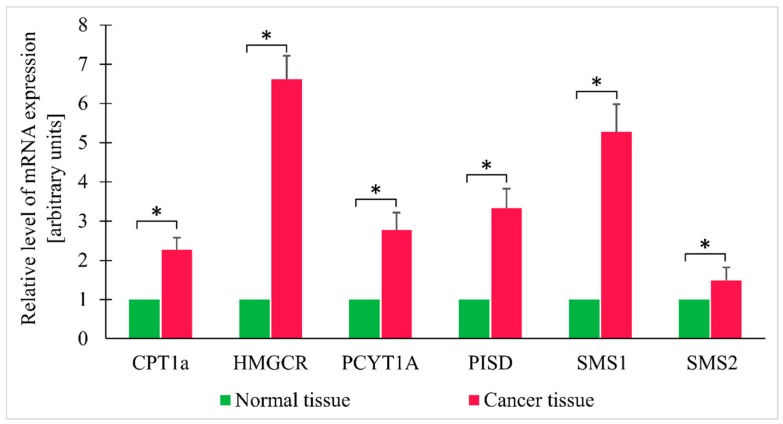
Relative expression levels of selected genes in normal and colorectal cancer tissue. Values are mean ± SEM; * *p* < 0.05, *n* = 25; CPT1a—Carnitine palmitoyltransferase I; HMGCR—HMG-CoA reductase; PCYT1A—choline-phosphate cytidylyltransferase A; PISD—phosphatidylserine decarboxylase; SMS1 - sphingomyelin synthase 1; SMS2; sphingomyelin synthase 2.

Since the findings reported above pointed to likely increase in TG oxidation and enhanced synthesis of membrane lipids in cancer tissue, the expressions of genes encoding key enzymes involved in FA oxidation (CPT1a—carnitine palmitoyl-CoA transferase 1a), cholesterol synthesis (HMGCR—HMG-CoA reductase), PC synthesis (PCYT1A—choline-phosphate cytidylyltransferase A), sphingomyelin ((sphingomyelin synthase 1 and 2—SMS1 and SMS2), and phosphatidylethanolamine (phosphatidylserine decarboxylase—PISD) were determined as well. Cancer tissue turned out to contain elevated levels of mRNA for all the enzymes mentioned above (Figure 5).

## 4. Discussion

In this study, we used ^1^H-NMR and GC-MS to identify differences in the lipid group and fatty acid profiles of CRC and normal tissue. Compared to normal tissue, CRC tissue contained higher levels of lipids serving as components of plasma membranes, such as phospholipids, sphingomyelin, and cholesterol, and had a lower content of TGs, the main source of energy in the cell. In line with these findings, CRC tissue contained also elevated levels of PUFAs and SFAs, the main components of polar membrane lipids, and lower levels of MUFAs, the primary component of TGs. The observation on elevated levels of PUFAs and SFAs and reduced levels of MUFAs (especially 18:1) in CRC tissue is consistent with the results of our previous research [28] and studies conducted by some other authors [18]. However, the results of the studies analyzing FA profile of colorectal cancer are inconclusive, as some researchers observed elevated levels of n-6 PUFAs and reduced levels of n-3 PUFAs in phospholipids from CRC tissues [29], whereas others reported opposite results, i.e., increased levels of n-3 PUFAs and decreased levels of n-6 PUFAs in the tumor tissue [30]. Our recent study demonstrated that CRC cells not only overexpress PUFA elongases and desaturases but also preferentially uptake PUFAs from their environment [31]. The reason for no difference in the content of LA and only slight difference in the content of ALA between normal and cancer tissue (Table 3), may be an overexpression of enzymes involved in the metabolism of these essential PUFAs, elongases, and desaturases, that we have reported recently [31]. Thus, we can hypothesize that the majority of LA and ALA pool are converted into other polyunsaturated fatty acids, such as DHA, EPA, and ARA, which levels are significantly elevated (Table 3). The present study suggest that this observation might be related to the fact that PUFAs are essential for the formation of cell membrane phospholipids during rapid proliferation of cancer cells. Furthermore, we showed that the changes in FA composition of CRC tissue were associated with alterations of complex lipid profiles in cancer cells, including polar lipids and TGs. Taken altogether, these results suggest that CRC cells are characterized by an intensive synthesis of polar lipids and rapid degradation of TGs for energy production. This hypothesis is also supported by concomitant overexpression of genes encoding enzymes associated with mitochondrial lipid oxidation (CPT1a) and synthesis of membrane lipids (HMGCR, PCYT1A, SMS1, SMS2, and PISD). FA derived from phospholipids by phospholipase A1 and A2 can also be transported by CPT1a into mitochondria and oxidized. However, increased content of phospholipids and decreased content of TG in cancer tissue suggest that FA derived from TG are preferentially used for β-oxidation in CRC tissue. Aside from the distinct role they play in cell membrane formation, a crucial process for rapidly proliferating cancer cells, many polar lipids and PUFAs, as well as their metabolites, e.g., fatty acids, eicosanoids, leukotrienes, phosphoinositides, and sphingolipids, act as signaling molecules. These signaling lipids have been implicated in tumor progression and metastasis [32].

In previous studies using ^1^H-NMR-based metabolic profiling, elevated levels of phospholipid precursors, choline, phosphocholine and phosphoethanolamine, were found in both human CRC tissues [33] and CRC CT26 cell line [34]. In the LC-MS/MS study conducted by Moro et al., the levels of SM in breast cancer were significantly higher than in peritumor tissue and normal tissue [35]. Alkaline sphingomyelinase converts SM to ceramide, a molecule that triggers cell apoptosis. The activity of alkaline sphingomyelinase in CRC tissue was shown to be significantly reduced, which might be a reason behind the elevated SM content; furthermore, the downregulation of alkaline sphingomyelinase might also lead to a decrease in ceramide levels, prevent apoptosis, and, hence, promote CRC development [2]. However, to the best of our knowledge, our present study was the only one to analyze the SM content in CRC tissue. In the GC×GC/TOF-MS-based metabolomics study conducted by Mal et al., cancer tissues were shown to contain elevated levels of cholesterol; aside from being a plasma membrane component, the latter is also a precursor of steroid hormones and bile acids [4]. Hence, the authors of the study mentioned above speculated that the products of cholesterol, especially bile acids, might act as tumor promoters or carcinogens in CRC [4]. Other researchers found elevated levels of triacylglycerols in CRC tissues [23,36] using ^1^H HR-MAS NMR spectroscopy. Similar to our present study, Mirnezami et al. [23] reported reduced ^1^H HR-MAS NMR spectroscopy signals for some FA groups, including CH3 (ppm 0.89), -(CH2)n (ppm 1.27), -CH2HC=C (ppm 2.02), and HC=CH- (ppm 5.36). While our findings are consistent with the results mentioned above, it needs to be emphasized that other authors used ^1^H-NMR to examine whole tissue extracts, and hence, were able to detect only some lipids, such as triacylglycerols, cholesterol and phospholipid precursors, such as choline, phosphocholine, and phosphoethanolamine. In our present study, in turn, the NMR analysis was preceded by lipid extraction [37], which enabled us to detect a much larger group of lipids and to analyze the composition of various lipid groups comprehensively. By contrast to our results, as well as the above discussed studies, the very recent study by Wang et al. [38] did not show significant difference in total lipids, as well as most of lipid groups between CRC tissue and adjacent normal mucosa. Concerning total lipids, it is difficult to explain this discrepancy between our, and their results, however, our method for evaluation the total lipid content in tissue is very simple—the lipids were extracted from tissue samples by proven and known for many years Folch method [26] and then weighted. Thus, we believe that the probability of incorrect measurement by our method is very low. In turn, it is impossible to compare our and Wang’s [38] lipid groups measurement results, since these authors present their results as pmol/µg of protein, whereas we present the NMR results as signal intensity normalized on 1 mg of lipids, so our results rather show the profile of lipid groups, that is the share of individual lipid groups in the entire lipid pool.

Many previous studies documented alterations in lipid metabolism in cancer tissues and demonstrated that lipid profile depends on the type of cancer [8] and its stage [23,39]. Most authors reported overexpression of lipogenic enzymes in cancer tissues or cells [17]. The overexpression of enzymes from this group, specifically FASN [40] and SCD1 [41] involved in the synthesis of oleic acid, the main component of triglycerides, was also found in CRC. These findings imply that the contents of lipids, including triglycerides, in cancer tissues should be elevated. Paradoxically, however, we found reduced levels of total lipids, especially TG, in CRC. A similar phenomenon was also reported by other authors [23,36]. In our opinion, the decrease in total lipid content in CRC tissue might be primarily a consequence of the substantial reduction of TG levels, probably due to enhanced oxidation. This hypothesis seems to be supported by the results published by Zaytseva et al. [42]; according to those authors, enhanced de novo lipogenesis and overexpression of FASN stimulated cellular respiration in CRC. Based on those findings, they postulated that endogenously synthesized lipids might serve as a fuel for fatty acid oxidation, especially during metabolic stress, and maintain energy homeostasis. Consistent with the above statement, we found elevated levels of carnitine palmitoyltransferase mRNA in CRC tissues (Figure 5). Carnitine palmitoyltransferase is involved in FA transport into mitochondria and limits the whole process of FA beta-oxidation, and overexpression of this enzyme was found previously by other authors in CRC cells cultured in vitro [43].

The results of many studies suggest that systemic alterations of lipid metabolism and excess of lipids in the diet may increase the risk of CRC [3,44]. Additionally, obesity seems to predispose to colorectal carcinogenesis [8]. Indeed, only four of our patients had a BMI below 25 kg/m^2^. Lipids released from adipocytes can be transferred to cancer cells which use them as a substrate for energy production through β-oxidation [3]. In our opinion, a better insight into the systemic alterations of lipid metabolism in CRC patients, and especially into the alterations within the tumor tissue, would facilitate the development of novel therapeutic strategies. Furthermore, some serum lipids might theoretically serve as CRC markers [28]. Despite progress in the diagnostics and treatment of cancer, earlier detection of the disease and development of novel targeted therapies, many malignancies still do not respond adequately to currently available regimens. Considering the likely role of lipid metabolism in cancer progression, one of the directions of further research should be the identification and synthesis of bioactive compounds that might modulate this process either directly or indirectly. With no doubt, the optimization of CRC treatment requires clinical trials combining classic chemotherapeutic agents with bioactive modulators of lipid metabolism [3]. The relatively small cohort was a limitation of our study, and did not allow for in-depth examination of dependencies between tumor lipid profiles nor serum lipidogram of patients and parameters such as tumor localization, disease stage or gender. Another limitation of this study could be heterogeneity of the tumors that may affect their lipid composition. However, the standard error of the mean for each of the tumor components were relatively low supporting homogeneity. Moreover, when we analyzed associations between lipid composition and tumor stroma ratio we found significant positive correlation only in case of 16:1 (r = 0.55; *p* < 0.05). Again, this may be due to small sample size. Thus the future studies on relations between parameters such as localization, disease stage or tumor histology in larger groups of patients are needed. But even with this sample size, the statistical significance of differences between normal and tumor tissue was quite robust and, therefore, convincing. LC-MS analysis allows for detection of more lipid compounds, than our method, but the advantage of our research is simultaneous analysis of main lipid groups by NMR and analysis of the main components of lipids - fatty acids by GC-MS. Such approach allows to obtain the profile main lipid groups together with fatty acid profile from small amounts of tissue that can be collected from patients during operation, and provides a lot of information about changes of lipid composition in CRC tissue.

## 5. Conclusions

In conclusion, this study showed that CRC tissue had lower levels of total lipids, contained fewer triglycerides and more membrane building polar lipids and cholesterol than normal large intestinal mucosa. This lipid rearrangement is probably associated with the rapid synthesis of plasma membranes in proliferating cancer cells and degradation of TGs as an energy substrate; however, this hypothesis needs to be verified empirically in future studies.

## Figures and Tables

**Table 1:** Biochemical and clinical characteristics of the study subjects. SEM—standard error of the mean; BMI—body mass index; CRP-hs—high-sensitivity C-reactive protein; HDL—high density lipoprotein; LDL—low density lipoprotein; T stage—tumor stage; UICC stage—Union for International Cancer Control stage.

Parameter	Mean ± SEM
Age (years)	69.8 ± 2.66
BMI (kg/m^2^)	28.4 ± 1.09
CRP-hs (mg/L)	3.53 ± 0.737
Total serum cholesterol (mg/dL)	155 ± 12.7
Triacylglycerols (mg/dL)	123 ± 11.2
HDL (mg/dL)	36.7 ± 2.57
LDL (mg/dL)	99.2 ± 8.44
Glucose (mg/dL)	105 ± 8.53
Albumin (g/dL)	3.11 ± 0.186
Total protein (g/dL)	6.10 ± 0.331
Stroma content in tumor (%)	22.9 ± 2.42
Connective tissue cells content in tumor (%)	15.1 ± 2.22
Inflammatory cells content in tumor (%)	7.79 ± 1.72
Epithelial cells content in tumor (%)	59.5 ± 4.51
Tumor-stroma ratio	5.36 ± 0.577
**Parameter**	**N**
Sex	
Male	16
Female	9
Location of primary tumor	
Cecum	9
Ascending colon	3
Transverse colon	2
Descending/proximal sigmoid colon	4
Rectosigmoid	3
Rectum	4
T stage	
T1	2
T2	9
T3	12
T4	2
UICC stage	
I	10
II	3
III	9
IV	3
Lymph node status	
N0	13
N1/N2	12

**Table 2:** Intensities of signals for various lipid groups obtained during ^1^H-NMR spectroscopic analysis.

L.p.	^1^H NMR signal	Chemical Shift * (ppm)	Normal Colon Tissue (Signal Intensity) Mean ± SEM	Colon Cancer Tissue (Signal Intensity) Mean ± SEM	*p*
1	-C18**H3** in total cholesterol	0.70	79.6 ± 6.93	141 ± 10.9	<0.001
2	-C26H3/-C27**H3** in total cholesterol	0.86	199 ± 13.8	256 ± 17.8	0.004
3	-C21**H3** in free cholesterol	0.93	167 ± 17.2	221 ± 16.1	0.011
4	-C19**H3** in free cholesterol	1.02	96.5 ± 9.25	150 ± 14.1	<0.001
5	-C19**H3** in esterified cholesterol	1.04	48.0 ± 11.9	58.4 ± 5.82	0.424
6	-C**H3** in fatty acyl chain	0.89	1813 ± 71.8	1444 ± 101	<0.001
7	-(C**H2**)n in fatty acyl chain	1.27	7444 ± 321	6315 ± 441	0.013
8	CHC**H2**CH2(CH2)- in fatty acyl chain	1.32	4810 ± 221	3078 ± 236	<0.001
9	-CO-C**H2**- in fatty acyl chain	2.32	1139 ± 49.3	769 ± 57.2	<0.001
10	-CO-CH2C**H2**- in fatty acyl chain	1.61	1272 ± 62.2	892 ± 70.0	<0.001
11	-HC=**CH**- in fatty acyl chain	5.36	725 ± 42.1	551 ± 71.4	0.028
12	-C**H2**HC=C in fatty acyl chain: 18:1	2.02	1013 ± 51.4	618 ± 52.9	<0.001
13	-C**H2**HC= in fatty acyl chain: 18:2n-6/20:4n-6	2.08	205 ± 17.0	163 ± 24.4	0.146
14	CHC**H2**CH= in fatty acyl chain: 18:2n-6	2.78	58.7 ± 7.21	40.7 ± 7.90	0.106
15	-CO-C**H2**- in fatty acyl chain: 22:6n-3	2.42	17.3 ± 1.44	21.8 ± 2.44	0.049
16	CHC**H2**CH= in fatty acyl chain: 20:4n-6/22:6n-3	2.84	66.8 ± 14.2	144 ± 30.7	0.022
17	-CH2-C**H2**-NH2 of PE	3.11	17.7 ± 2.41	46.0 ± 5.08	<0.001
18	C2**H** in glycerol backbone of PE	3.26	15.3 ± 1.85	27.3 ± 3.94	0.007
19	-N+(C**H3**)3 in SM head group	3.21	84.6 ± 7.53	141 ± 10.3	<0.001
20	-C**H2**N+(CH3)3 in SM head group	3.62	92.8 ± 6.76	161 ± 13.3	<0.001
21	-C**H2**CH2N+(CH3)3 in SM head group	4.25	97.0 ± 8.87	139 ± 13.7	0.005
22	-C**H2**N+(CH3)3 in PC head group	3.22	177 ± 71.8	1139 ± 338	<0.001
23	-N+(C**H3**)3 in PC head group	3.68	255 ± 23.7	495 ± 40.6	<0.001
24	>C3**H2** in glycerol backbone of PL	4.01	171 ± 18.2	309 ± 27.2	<0.001
25	-C2**H** in glycerol backbone of PL	5.24	38.3 ± 3.28	71.1 ± 7.43	<0.001
26	>C1**H2**/C3**H2** in glycerol backbone of TG	4.33	334 ± 20.9	137 ± 17.4	<0.001
27	-C2**H** in glycerol backbone of TG	5.28	134 ± 10.0	41.6 ± 6.11	<0.001
28	>C1**H2**/C3**H2** in glycerol backbone of TG and PL	4.16	368 ± 21.0	210 ± 18.7	<0.001

* Chemical shift for proton or protons in bold. PE—phosphatidylethanolamines; PL—phospholipids; SM—sphingomyelin; PC—phosphatidylcholines; TG—triacylglycerols.

**Table 3:** Contents (% of total FA) of main FAs and FA groups in normal and colorectal cancer tissue.

Fatty Acids	Normal	Cancer Tissue	*p*
16:0	22.0 ± 0.368	20.7 ± 0.318	0.017
18:0	7.14 ± 0.467	12.5 ± 0.567	<0.001
**Total SFA**	**32.6 ± 0.657**	**36.8 ± 0.519 ^a^**	**<0.001**
16:1	4.86 ± 0.353	3.34 ± 0.201	<0.001
18:1	45.0 ± 0.953	35.6 ± 1.07	<0.001
**Total MUFA**	**51.1 ± 1.15**	**40.6 ± 1.21 ^b^**	**<0.001**
18:2 (LA)	11.1 ± 0.398	11.2 ± 0.399	0.682
20:4 (ARA)	2.99 ± 0.349	6.73 ± 0.500	<0.001
**Total n-6 PUFA**	**15.2 ± 0.722**	**20.5 ± 0.719 ^a^**	**<0.001**
18:3 (ALA)	0.040 ± 0.004	0.050 ± 0.007	0.012
20:5 (EPA)	0.210 ± 0.025	0.450 ± 0.041	<0.001
22:6 (DHA)	0.450 ± 0.044	1.02 ± 0.059	<0.001
**Total n-3 PUFA**	**1.02 ± 0.082**	**2.11 ± 0.107 ^a^**	**<0.001**

^a^ Upregulated; ^b^ downregulated. Values are mean ± SEM. Bold represents main groups of fatty acids.

## Supplementary Materials

The following are available online at https://www.mdpi.com/2077-0383/9/4/1095/s1, Figure S1: Total lipid content in normal and colorectal cancer tissue, Figure S2: Example of a ^1^H-NMR spectrum for lipids isolated from healthy tissue and CRC tissue, Figure S3: Intensities of ^1^H-NMR signal for selected groups of lipids, Table S1: Primer sequence for RT-PCR, Table S2: Analysis of correlations between the ^1^H-NMR signal intensities of selected groups of lipids and the levels of fatty acids, and patients BMI, serum triacyclglycerols and total cholesterol, Table S3: Differences in ^1^H-NMR signal intensity of selected lipid groups and fatty acid levels in tumor tissue in various groups of CRC patients.
