# Supplementary material for: Decreased Triacylglycerol Content and Elevated Contents of Cell Membrane Lipids in Colorectal Cancer Tissue: A Lipidomic Study
Source: J Clin Med. 2020 Apr 12;9(4):1095. doi: 10.3390/jcm9041095 (PMC7230725; doi:10.3390/jcm9041095)
Supplement: Supplementary file 1 [file jcm-09-01095-s001.pdf]

**Table 1.** Primer sequence for RT-PCR. ACTB – beta-actin, HMGCR - HMG-CoA reductase, CPT1a - carnitine palmitoyltransferase I, CHKA - choline kinase alpha, CHKB - choline kinase beta, PCYT1A- choline-phosphate cytidylyltransferase A, CHPT1 - cholinephosphotransferase 1, PISD - phosphatidylserine decarboxylase, SMS1 - sphingomyelin synthase 1, SMS2- sphingomyelin synthase 2.

| Gene name | Forward sequence (from 5` to 3`) | Reverse sequence (from 5` to 3`) |
|---|---|---|
| ACTB | AGCACAGAGCCTCGCCTT | CATCATCCATGGTGAGCTGG |
| CPT1a | ATCAATCGGACTCTGGAAACGG | TCAGGGAGTAGCGCATGGT |
| HMGCR | TACCATGTCAGGGGTAC | CAAGCCTAGAGACATAAT |
| CHKA | CGGAAGTATCCCACCAAGAA | TCCCCAGAGGAAATGAGATG |
| CHKB | TGGTGCTAGAAAGCGTGATG | GCCGACTTGGGATGTACT |
| PCYT1A | GCAACCAGCTCCTTTTTCTG | GCAAACTCCCACAATGAGGT |
| CHPT1 | TCTGCTCTTTTATTGGGATGTTTG | CAACACAAAGACAATCACTAAAGC |
| PISD | ATCACTACCGCAACCTCAGCGA | TACCTGCTCCACCTCACAGTTC |
| SMS 1 | GCACTTTCCTGTTTCGTTCTC | TACAGCGTGCCAACTATGC |
| SMS 2 | GCATTTCCAGTGTGCTCCAAAGC | GTAACCGTGTGACCGCTGAAGA |

**Table 2.** Analysis of correlations between the $^{1}$H-NMR signal intensities of selected groups of lipids and the levels of fatty acids, and patients BMI, serum triacyclglycerols and total cholesterol.

| Lipid/lipid group | Correlation with BMI | | Correlation with serum triacylglycerols | | Correlation with serum total cholesterol | |
|---|---|---|---|---|---|---|
| | R | p | R | p | R | p |
| Free cholesterol | -0.101 | 0.662 | -0.116 | 0.588 | 0.099 | 0.644 |
| Phosphatidylethanolamines | 0.096 | 0.679 | -0.165 | 0.441 | -0.215 | 0.312 |
| Sphingomyelin | -0.066 | 0.776 | -0.259 | 0.222 | -0.024 | 0.910 |
| Phosphatidylcholines | -0.043 | 0.852 | -0.260 | 0.221 | -0.013 | 0.951 |
| Phospholipids | -0.071 | 0.758 | -0.302 | 0.152 | -0.139 | 0.517 |
| Triacylglycerols | -0.425 | 0.053 | -0.181 | 0.397 | -0.120 | 0.575 |
| 16:0 | 0.063 | 0.786 | -0.218 | 0.306 | 0.269 | 0.204 |
| 18:0 | 0.024 | 0.918 | 0.069 | 0.750 | -0.087 | 0.685 |
| Total SFA | -0.034 | 0.884 | -0.114 | 0.597 | 0.023 | 0.916 |
| 16:1 | 0.118 | 0.609 | 0.121 | 0.574 | 0.140 | 0.516 |
| 18:1 | 0.044 | 0.850 | 0.048 | 0.824 | -0.056 | 0.795 |
| Total MUFA | 0.024 | 0.918 | 0.060 | 0.780 | -0.044 | 0.838 |
| 18:2 (LA) | 0.054 | 0.815 | -0.155 | 0.470 | 0.194 | 0.363 |
| 20:4 (ARA) | -0.030 | 0.899 | 0.046 | 0.831 | -0.012 | 0.955 |
| Total n-6 PUFA | -0.028 | 0.903 | -0.005 | 0.982 | 0.053 | 0.806 |
| 18:3 (ALA) | -0.127 | 0.583 | -0.124 | 0.563 | -0.350 | 0.093 |
| 20:5 (EPA) | 0.208 | 0.364 | -0.246 | 0.247 | 0.012 | 0.956 |
| 22:6 (DHA) | -0.021 | 0.928 | -0.050 | 0.815 | 0.096 | 0.655 |
| Total n-3 PUFA | 0.079 | 0.733 | -0.109 | 0.612 | 0.054 | 0.803 |

R - Pearson's correlation coefficient, p - significance.

**Table 3.** Differences in $^{1}$H-NMR signal intensity of selected lipid groups and fatty acid levels in tumor tissue in various groups of CRC patients.

| Lipid/lipid group | CRC patients with T stage 1 and 2 Mean ± SEM | CRC patients with T stage 3 and 4 Mean ± SEM | p | Male CRC patients Mean ± SEM | Female CRC patients Mean ± SEM | p |
|---|---|---|---|---|---|---|
| Free cholesterol [a] | 163 ± 19.1 | 140 ± 20.4 | 0.437 | 130 ± 16.4 | 186 ± 22.4 | 0.053 |
| Phosphatidylethanolamines [a] | 31.9 ± 6.20 | 23.6 ± 5.07 | 0.309 | 23.9 ± 19.1 | 33.3 ± 6.81 | 0.259 |
| Sphingomyelin [a] | 181 ± 15.5 | 146 ± 19.8 | 0.201 | 150 ± 16.9 | 181 ± 20.9 | 0.271 |
| Phosphatidylcholines [a] | 563 ± 47.9 | 443 ± 60.3 | 0.149 | 461 ± 50.0 | 557 ± 69.6 | 0.272 |
| Phospholipids [a] | 81.1 ± 10.5 | 63.2 ± 10.2 | 0.240 | 64.6 ± 9.21 | 82.7 ± 12.3 | 0.251 |
| Triacylglycerols [a] | 45.3 ± 9.72 | 38.6 ± 8.01 | 0.603 | 40.6 ± 6.56 | 43.4 ± 12.8 | 0.837 |
| 16:0 [b] | 21.0 ± 0.485 | 20.5 ± 0.429 | 0.466 | 20.5 ± 0.462 | 21.0 ± 0.340 | 0.486 |
| 18:0 [b] | 12.3 ± 0.825 | 12.7 ± 0.777 | 0.728 | 12.5 ± 0.656 | 12.7 ± 1.07 | 0.867 |
| Total SFA [b] | 36.7 ± 0.778 | 36.9 ± 0.722 | 0.857 | 36.8 ± 0.593 | 36.9 ± 1.04 | 0.907 |
| 16:1 [b] | 3.38 ± 0.330 | 3.31 ± 0.259 | 0.863 | 3.41 ± 0.266 | 3.23 ± 0.313 | 0.679 |
| 18:1 [b] | 35.4 ± 1.57 | 35.9 ± 1.50 | 0.828 | 35.8 ± 1.24 | 35.5 ± 2.08 | 0.896 |
| Total MUFA [b] | 40.0 ± 1.81 | 40.7 ± 1.69 | 0.778 | 40.5 ± 1.46 | 40.0 ± 2.26 | 0.851 |
| 18:2 (LA) [b] | 12.0 ± 0.651 | 10.6 ± 0.453 | 0.087 | 11.0 ± 0.547 | 11.6 ± 0.553 | 0.519 |
| 20:4 (ARA) [b] | 6.58 ± 0.579 | 6.85 ± 0.787 | 0.793 | 6.77 ± 0.640 | 6.66 ± 0.847 | 0.922 |
| Total n-6 PUFA [b] | 21.0 ± 1.05 | 20.1 ± 1.00 | 0.542 | 20.4 ± 0.930 | 20.8 ± 1.18 | 0.765 |
| 18:3 (ALA) [b] | 0.061 ± 0.011 | 0.050 ± 0.008 | 0.424 | 0.060 ± 0.009 | 0.046 ± 0.008 | 0.304 |
| 20:5 (EPA) [b] | 0.466 ± 0.077 | 0.455 ± 0.045 | 0.804 | 0.499 ± 0.052 | 0.376 ± 0.062 | 0.156 |
| 22:6 (DHA) [b] | 1.00 ± 0.091 | 1.03 ± 0.080 | 0.845 | 1.00 ± 0.067 | 1.05 ± 0.117 | 0.678 |
| Total n-3 PUFA [b] | 2.11 ± 0.190 | 2.10 ± 0.125 | 0.957 | 2.12 ± 0.126 | 2.08 ± 0.204 | 0.840 |
| Number of samples | 11 | 14 | | 16 | 9 | |

[a] Signal intensity form $^{1}$H-NMR (relative area), [b] % of total fatty acid content, p - significance from two-tailed t-test.

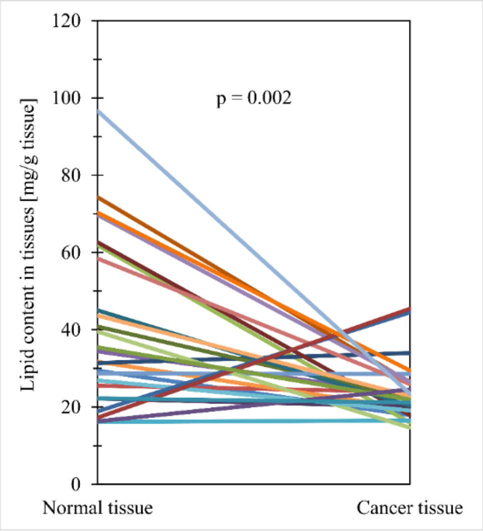


**Figure 1.** Total lipid content in normal and colorectal cancer tissue. Each line represents individual patient; $n = 25$.

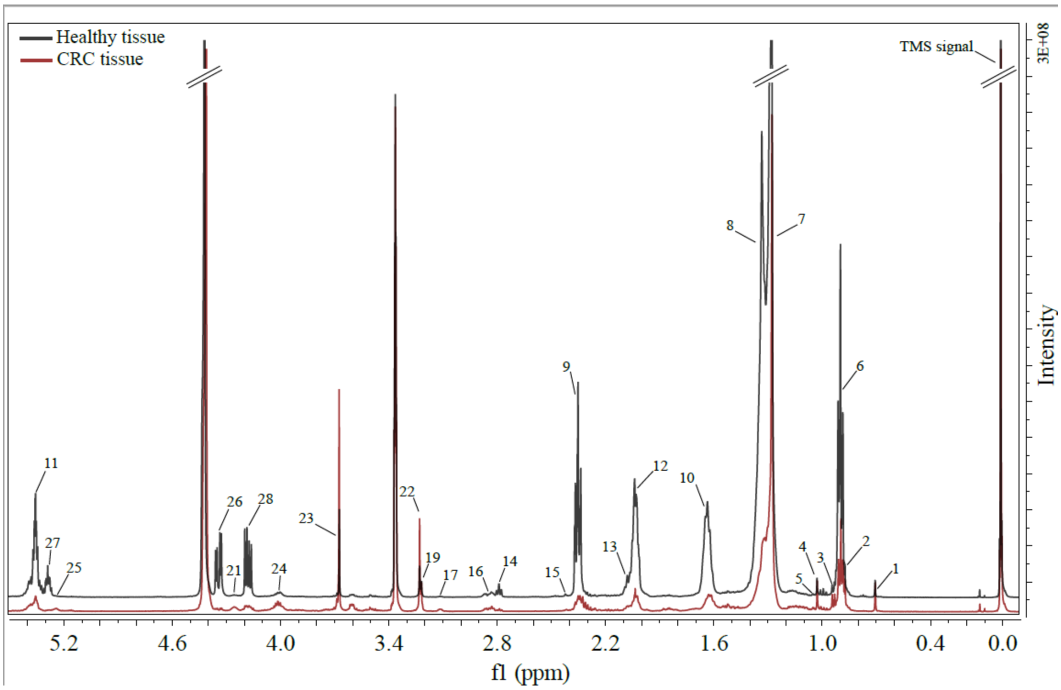


**Figure 2.** Example of a $^1$H-NMR spectrum for lipids isolated from healthy tissue and CRC tissue. 1 - C18**H3** in total cholesterol; 2 -C26**H3**/-C27**H3** in total cholesterol; 3 -C21**H3** in free cholesterol; 4 - C19**H3** in free cholesterol; 5 -C19**H3** in esterified cholesterol; 6 - -**CH3** in fatty acyl chain; 7 - -(**CH2**)n in fatty acyl chain; 8 - CHC**H2**CH2(CH2)- in fatty acyl chain; 9 - -CO-C**H2**- in fatty acyl chain; 10 - -CO-CH2C**H2**- in fatty acyl chain; 11 -**HC=CH**- in fatty acyl chain; 12 -C**H2**HC =C in fatty acyl chain: 18:1; 13 -C**H2**HC= in fatty acyl chain: 18:2n-6/20:4n-6; 14 CHC**H2**CH= in fatty acyl chain: 18:2n-6; 15 -CO-C**H2**- in fatty acyl chain: 22:6n-3; 16 CHC**H2**CH = in fatty acyl chain: 20:4n-6/22:6n-3; 17 -CH2-C**H2**-NH2 of PE; 18 C2**H** in glycerol backbone of PE; 19 -N+(C**H3**)3 in SM head group; 20 -C**H2**N+(CH3)3 in SM head group; 21 -C**H2**CH2N+(CH3)3 in SM head group; 22 -C**H2**N+(CH3)3 in PC head group; 23 -N+(C**H3**)3 in PC head group; 24 >C3**H2** in glycerol backbone of PL; 25 -C2**H** in glycerol backbone of PL; 26 >C1**H2**/C3**H2** in glycerol backbone of TG; 27 -C2**H** in glycerol backbone of TG; 28 >C1**H2**/C3**H2** in glycerol backbone of TG and PL.

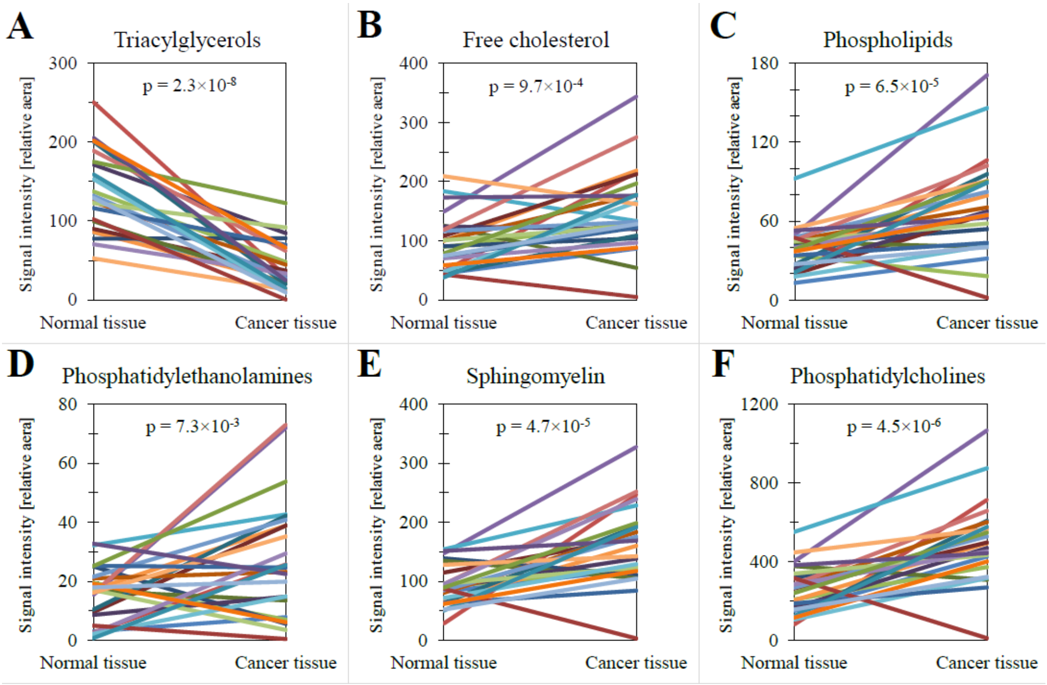


**Figure 3.** Intensities of $^{1}$H-NMR signal for selected groups of lipids: (A) triacylglycerols; (B) free cholesterol; (C) phospholipids; (D) phosphatidylethanolamines; (E) sphingomyelin; (F) phosphatidylcholines. Each line represents individual patient; n=25.
